# Disrupted Asymmetry of Inter- and Intra-Hemispheric Functional Connectivity at Rest in Medication-Free Obsessive-Compulsive Disorder

**DOI:** 10.3389/fnins.2021.634557

**Published:** 2021-06-09

**Authors:** Cuicui Jia, Yangpan Ou, Yunhui Chen, Jidong Ma, Chuang Zhan, Dan Lv, Ru Yang, Tinghuizi Shang, Lei Sun, Yuhua Wang, Guangfeng Zhang, Zhenghai Sun, Wei Wang, Xiaoping Wang, Wenbin Guo, Ping Li

**Affiliations:** ^1^Department of Psychiatry, Qiqihar Medical University, Qiqihar, China; ^2^National Clinical Research Center for Mental Disorders, and Department of Psychiatry, The Second Xiangya Hospital of Central South University, Changsha, China; ^3^Department of Psychiatry, Baiyupao Psychiatric Hospital of Harbin, Harbin, China; ^4^Department of Radiology, The Second Xiangya Hospital of Central South University, Changsha, China; ^5^Department of Radiology, The Third Affiliated Hospital of Qiqihar Medical University, Qiqihar, China; ^6^Department of Library, Qiqihar Medical University, Qiqihar, China

**Keywords:** obsessive-compulsive disorder, functional magnetic resonance imaging, resting-state, parameter of asymmetry, support vector machine

## Abstract

Disrupted functional asymmetry of cerebral hemispheres may be altered in patients with obsessive-compulsive disorder (OCD). However, little is known about whether anomalous brain asymmetries originate from inter- and/or intra-hemispheric functional connectivity (FC) at rest in OCD. In this study, resting-state functional magnetic resonance imaging was applied to 40 medication-free patients with OCD and 38 gender-, age-, and education-matched healthy controls (HCs). Data were analyzed using the parameter of asymmetry (PAS) and support vector machine methods. Patients with OCD showed significantly increased PAS in the left posterior cingulate cortex, left precentral gyrus/postcentral gyrus, and right inferior occipital gyrus and decreased PAS in the left dorsolateral prefrontal cortex (DLPFC), bilateral middle cingulate cortex (MCC), left inferior parietal lobule, and left cerebellum Crus I. A negative correlation was found between decreased PAS in the left DLPFC and Yale–Brown Obsessive-compulsive Scale compulsive behavior scores in the patients. Furthermore, decreased PAS in the bilateral MCC could be used to distinguish OCD from HCs with a sensitivity of 87.50%, an accuracy of 88.46%, and a specificity of 89.47%. These results highlighted the contribution of disrupted asymmetry of intra-hemispheric FC within and outside the cortico-striato-thalamocortical circuits at rest in the pathophysiology of OCD, and reduced intra-hemispheric FC in the bilateral MCC may serve as a potential biomarker to classify individuals with OCD from HCs.

## Introduction

Asymmetry is considered when one side of the brain is structurally or functionally different from the other side (Zach et al., 2016). Structural and functional asymmetry of cerebral hemispheres exists in the healthy population (Crow et al., 1989), which may be due to hereditary, developmental, evolutionary, pathological, and epigenetic factors (Samara and Tsangaris, 2011). Healthy individuals can benefit from normal asymmetries. However, the asymmetries are disrupted in some mental disorders, such as schizophrenia (Federspiel et al., 2006), attention-deficit/hyperactivity disorder (Shaw et al., 2009), somatization disorder (Su et al., 2020), and autism (Herbert et al., 2005). Patients with obsessive-compulsive disorder (OCD) also displayed anomalous brain asymmetries (Boedhoe et al., 2017). Moreover, patients with OCD and their siblings exhibited leftward asymmetry of cortical thickness in the anterior cingulate cortex, and this abnormal asymmetry was positively correlated with compulsive subscale scores (Peng et al., 2015). Pediatric and adult patients with OCD both showed significantly altered brain volume asymmetries (Wang et al., 2020). For example, pediatric OCD displayed brain volume asymmetry in the pallidum and thalamus (Kong et al., 2020). In addition, reduced interhemispheric functional connectivity (FC) within (i.e., orbital frontal cortex and thalamus) and outside (i.e., inferior occipital gyrus and precentral gyrus/postcentral gyrus) the cortico-striato-thalamocortical (CSTC) circuits in OCD has been discovered by the authors’ group (Jia et al., 2020). Taken together, structural and functional asymmetry of cerebral hemispheres exists in patients with OCD.

Given that the corpus callosum connects functional interaction and cooperation between two hemispheres (Schüz and Preissl, 1996; Doron and Gazzaniga, 2008), each individual brain region has FCs related to inter- and intra-hemispheric connections, thus contributing to the information communication of cognition and behavior (Zhu et al., 2019). Despite significant advances in OCD research (Jia et al., 2020), whether the asymmetric functional disruption of OCD resulting from inter- and/or intra-hemispheric FCs and the extent of these FCs contribute to abnormal functional asymmetry in OCD remain unclear.

We have used the voxel-mirrored homotopic connectivity (VMHC) method to investigate the interhemispheric coordination at rest in OCD in a previous study (Jia et al., 2020), whereas the quantitative parameter of asymmetry (PAS) was used to calculate an abnormal voxel and other voxels from inter- and/or intra-hemispheric asymmetries (Zhu et al., 2018) in this study. Both the PAS and VMHC methods involve interhemispheric FC between the two hemispheres of the brain. However, they are used to assess different aspects of interhemispheric FC. VMHC is applied to assess FC between a given voxel in one hemisphere and its corresponding voxel in the other hemisphere, whereas interhemispheric FC to quantify PAS is utilized to measure a given voxel in one hemisphere and all voxels in the other hemisphere. PAS is a voxel-wise approach that is unaffected by preselected regions of interest (ROIs) and thresholds, thus minimizing the potential confounding effects of structural asymmetries and selection bias through ROIs (Zhu et al., 2018). It has been widely used in exploring inter- and/or intra-hemispheric asymmetries in patients with schizophrenia and their unaffected siblings, individuals at ultra-high risk for psychosis, major depressive disorder (Ding et al., 2021), and somatization disorder (Zhu et al., 2018, 2019; Su et al., 2020).

In the current research, functional asymmetries of the whole-brain regions at rest in medication-free patients with OCD were investigated using the PAS approach to address the issue whether the asymmetric disruption of OCD results from inter- and/or intra-hemispheric FCs. Based on previous studies, patients with OCD were hypothesized to have significantly abnormal inter- and/or intra-hemispheric FCs within and outside the CSTC circuits. In addition, disrupted asymmetries may be related to clinical characteristics and can be used as potential neurobiological markers to distinguish OCD from healthy controls (HCs).

## Experimental Procedures

### Participants

Forty subjects (27 males and 13 females) with OCD were recruited from the Fourth Affiliated Hospital of Qiqihar Medical University and Qiqihar Mental Health Center, China. The diagnosis was based on the Structured Clinical Interview for DMS-IV (SCID), patient version (First et al., 1996). The Yale–Brown Obsessive-Compulsive Scale (Y-BOCS), Hamilton Anxiety Rating Scale (HAMA), and 17-item Hamilton Depression Rating Scale (HAMD) were used to assess clinical symptoms of OCD. A total of 22 patients had a history of antipsychotic, anti-obsessive medication, or antidepressant use, whereas 18 patients were drug naive. None of the patients took any psychotropic drugs for at least 4 weeks before brain-image acquisition. Thirty-eight HCs (25 males and 13 females) that matched with the patients in gender, education, and age were enrolled from the local community by using the SCID, non-patient version (First et al., 2001). All individuals were right-handed, were Han Chinese, and had the same inclusion criteria as follows: (1) 16–50 years old, (2) no serious physical disease or neurological or psychiatric illness, (3) no drug or alcohol dependence, (4) no pregnancy, (5) no contraindication for an MRI scan, and (6) no movement distance of more than 2 mm or rotation angle of more than 2°. HCs who had a first-degree relative with any mental disorders were excluded.

This study was approved by the Research Ethics Committee of Qiqihar Medical University. The participants were informed of the study procedures, and they signed a written informed consent.

### MRI Data Acquisition and Preprocessing

For all participants, resting-state functional magnetic resonance imaging (rs-fMRI) using a 3.0-Tesla GE 750 Signa-HDX scanner (General Electric Healthcare, Waukesha, WI, United States) with a 12-channel standard head coil was conducted at The Third Affiliated Hospital of Qiqihar Medical University. All individuals were instructed to use foam pads and earplugs to reduce the effect of scanner noise, remain in the supine position, close their eyes, relax, stay awake, and remain motionless (especially the head). The rs-fMRI data were obtained *via* an echo-planar imaging (EPI) sequence: axial slices = 33, repetition time = 2000 ms, echo time = 30 ms, slice thickness = 3.5 mm, inter-slice gap = 0.6 mm, flip angle = 90°, field of view = 200 × 200 mm^2^, data matrix = 64 × 64, and 240 volumes (8 min) in total. None of the subjects displayed clinically significant brain structural lesions.

Data were preprocessed with the Data Processing & Analysis for Brain Imaging (DPABI) software (Yan et al., 2016). The first 10 functional volumes were discarded to ensure a steady initial signal and adapt to the environment. Slice time correction and head motion correction were performed for the remaining 230 EPI images. Images were then spatially normalized to a standard Montreal Neurological Institute space and resampled to 3 mm × 3 mm × 3 mm. Afterward, the normalized images were smoothed with a 4-mm full-width half-maximum isotropic Gaussian kernel. Subsequently, the signal was linearly detrended and band-pass filtered (0.01–0.08 Hz) to reduce the covariate effect of high-frequency physiological noises and low-frequency drifts. The signals from the cerebrospinal fluid, white matter, and 24 motion parameters were used as nuisance covariates. The identification of “bad” timepoints was scrubbed using a threshold of 0.2 mm of framewise displacement (FD) and one back and two subsequent neighbors (Power et al., 2012), and the mean FD for each subject was calculated.

### Calculation of the PAS Values

Parameter of asymmetry analysis was conducted by using codes. The data were analyzed using the voxel-wise whole-brain analysis to calculate the correlation coefficients between a given voxel and the other voxels from the same hemisphere (intra-hemispheric coefficient) or the opposite hemisphere (interhemispheric coefficient). The intra-hemispheric or interhemispheric FC of this voxel was acquired by taking the mean of intra-hemispheric or interhemispheric coefficients. We used the whole-brain mask to calculate the PAS. Small correlation coefficients between voxels may show the confounding effects in the asymmetric analyses. Therefore, weak correlations were removed with a correlation coefficient threshold (*r* > 0.2) for PAS calculation (Wang et al., 2014; Liu et al., 2015). In addition, only positive correlations were used in the PAS calculation due to ambiguous explanations to negative correlations.

In PAS calculation, only the positive correlations were used, as reported previously (Zhu et al., 2018), and the mean coefficients were transformed to *z*-values (Buckner et al., 2009). The formula for PAS (Zhu et al., 2018) was as follows:

$$\text{PAS}={\text{FC}}_{\text{inter}}-{\text{FC}}_{\text{intra}},$$

FC_inter_, interhemispheric FC and FC_intra_, intra-hemispheric FC. When the PAS scores were positive, the asymmetry was primarily caused by the interhemispheric FC. When the PAS scores were negative, the asymmetry was mainly caused by the intra-hemispheric FC.

### Statistical Analysis

The demographic and clinical data were analyzed using two-sample *t*-tests and the *X*^2^ test with SPSS version 23.0 (SPSS Inc., Chicago, IL, United States). In each group, the one-sample *t*-test of PAS was performed in the DPABI software; *p* < 0.05 corrected by Gaussian random field theory (voxel significance: *p* < 0.001, cluster significance: *p* < 0.05) was the significance level. Group differences of PAS were conducted with voxel-wise two-sample *t*-tests in the DPABI software with *p* < 0.05 corrected by Gaussian random field (GRF). The mean FD values and age were used as covariates to reduce the potential effects of these variables.

Pearson’s correlation analyses between the mean PAS scores and clinical variables were conducted in OCD. The Bonferroni-corrected significance level was set at *pc* < 0.05. Twenty-one Pearson’s correlations were performed (seven brain regions × three scores of the Y-BOCS). The Bonferroni-corrected significance level was set at *pc* < 0.05/21.

### Support Vector Machine Analysis

Support vector machine (SVM) is a well-known and popular supervised machine learning technique with higher accuracy and precision. It effectively defines a set of information as well as the functions of different brain regions to find a maximum boundary delimiter to classify the data, which can differentiate patients from HCs using structural and/or functional neuroimaging data such as rs-fMRI (Jia et al., 2020). The orientation of the hyperplane is as far away from the nearest data point of each class as possible. These closest points are called support vectors (Huang et al., 2018). The exploratory analysis was applied to test whether abnormal PAS could be used to classify patients with OCD and HCs. The process of SVM classification consists of three steps: preparing data for classifier training, training and testing, and performance evaluation (Orrù et al., 2012). The “leave-one-out” cross-validation approach was used to acquire the highest sensitivity and specificity, which involved excluding a single subject from each group and using the remaining subjects to train the classifier (Bu et al., 2019). A detailed description of the SVM can be found in a previous study (Liu et al., 2013).

## Results

### Demographic and Clinical Data

The demographic and clinical data of participants are displayed in Table 1. A total of 40 medication-free patients with OCD and 38 HCs consented to enroll in this research. The two groups did not differ in terms of age (*t* = 0.05, *p* = 0.71), gender (*X*^2^ = 0.32, *p* = 1.00), educational level (*t* = 0.50, *p* = 0.83), and FD (*t* = 1.25, *p* = 0.13). Among the clinical characteristics, significant group differences were found in Y-BOCS (*t* = 25.27, *p* < 0.01), HAMD (*t* = 9.04, *p* < 0.01), and HAMA (*t* = 9.00, *p* < 0.01).

**TABLE 1:** Demographic and clinical characteristics of participants.

	**OCD patients (*n* = 40)**	**HCs (*n* = 38)**	***X*^2^/*t***	***p***
Age (years)	27.28 ± 8.16	27.18 ± 8.33	0.05	0.71
Sex (male/female)	27/13	25/13	0.32	1.00
Education (years)	13.40 ± 2.87	13.74 ± 3.03	−0.50	0.83
Illness duration (months)	66.68 ± 75.54			
Y-BOCS total score	24.90 ± 5.73	1.13 ± 0.88	25.27	<0.01
Y-BOCS obsessive thinking	12.85 ± 4.25	0.37 ± 0.49	17.98	<0.01
Y-BOCS compulsive behavior	12.05 ± 4.62	0.74 ± 0.72	14.92	<0.01
HAMD	8.05 ± 4.40	1.45 ± 0.95	9.04	<0.01
HAMA	10.83 ± 6.55	1.16 ± 1.00	9.00	<0.01
FD	0.04 ± 0.02	0.03 ± 0.01	1.25	0.13
Time points scrubbed out	1.13 ± 2.256	1.00 ± 2.418	0.25	0.95

*OCD, obsessive-compulsive disorder; Y-BOCS, Yale–Brown Obsessive-Compulsive Scale; HAMD, 17-item Hamilton Depression Rating Scale; HAMA, Hamilton Anxiety Rating Scale; FD, framewise displacement. Variables of age, education, Y-BOCS total score, subscales score, HAMD score, HAMA score, and FD were tested by two sample *t*-tests, and the results were indicated by *t*-values. Categorical data such as gender was tested using a Chi-squared test, and the result was indicated by *X*^2^.*

### Group Differences in PAS

**FIGURE 1:**
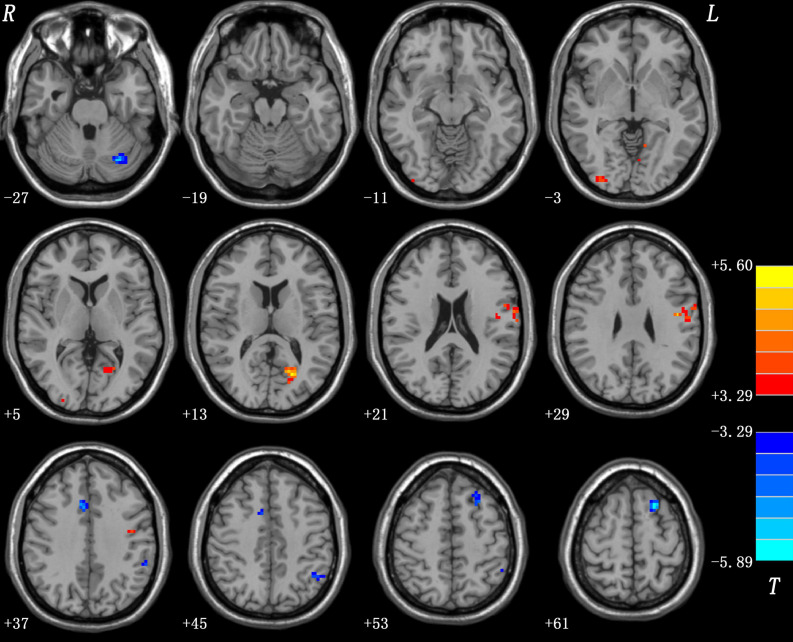
Differences in PAS scores between patients with OCD and HCs. Increased and decreased PAS scores were presented on red and blue colors, respectively. The color bar represents the *t*-values of the group analysis. OCD, obsessive-compulsive disorder; HCs, healthy controls; PAS, parameter of asymmetry.

Patients with OCD displayed significantly increased PAS in the left posterior cingulate cortex (PCC), left precentral gyrus/postcentral gyrus, and right inferior occipital gyrus, and decreased PAS in the left dorsolateral prefrontal cortex (DLPFC), bilateral middle cingulate cortex (MCC), left inferior parietal lobule (IPL), and left cerebellum Crus I compared to HCs (as shown in Figure 1 and Table 2). The details of PAS values in the OCD and HC groups are presented in Supplementary Table 1. The results of one-sample *t*-tests of PAS in each group are shown in Supplementary Figure 1.

**TABLE 2:** The differences of PAS scores between patients with OCD and HCs.

**Cluster location**	**Peak (MNI)**			**Number of voxels**	***t*-Value**
	***x***	***y***	***z***		
Left posterior cingulate cortex	–21	–66	9	61	5.5996
Left precentral gyrus/postcentral gyrus	–45	–9	30	92	5.1088
Right inferior occipital gyrus	33	–90	–6	30	4.9227
Left dorsolateral prefrontal cortex	–21	15	60	58	–5.8926
Bilateral middle cingulate cortex	9	18	36	42	–4.8529
Left inferior parietal lobule	–48	–54	45	56	–4.0077
Left cerebellum Crus I	–30	–72	–27	38	–5.1077

*The significance level was set at *p* < 0.05 for multiple comparisons corrected by Gaussian random field (GRF) theory (voxel significance: *p* < 0.001, cluster significance: *p* < 0.05). Age and the mean FD values were used as covariates to minimize the potential effects of these variables. PAS, parameter of asymmetry; OCD, obsessive-compulsive disorder; HCs, healthy controls.*

### Correlation Analysis

**FIGURE 2:**
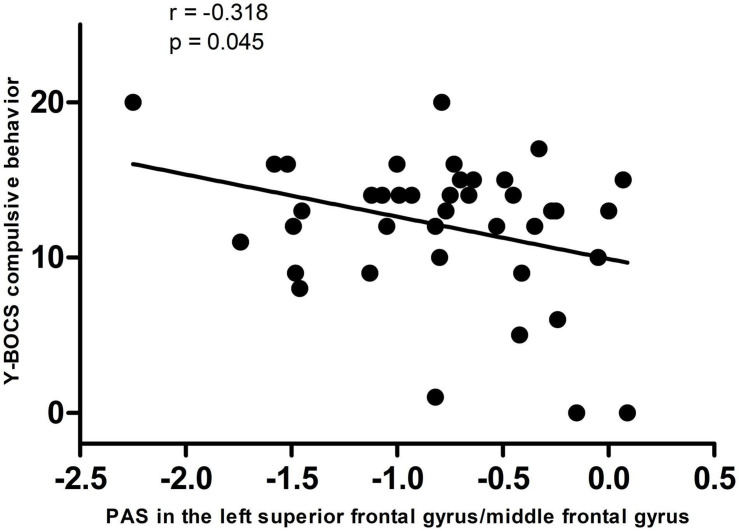
A negative correlation between PAS scores in the left dorsolateral prefrontal cortex and Y-BOCS compulsive behavior scores in the patients. PAS, parameter of asymmetry; Y-BOCS, Yale–Brown Obsessive-Compulsive Scale.

A significantly negative correlation was observed between the PAS score in the left DLPFC and Y-BOCS compulsive behavior scores in OCD (*r* = −0.318, *pc* = 0.045, Bonferroni corrected, Figure 2).

### SVM Analysis

**FIGURE 3:**
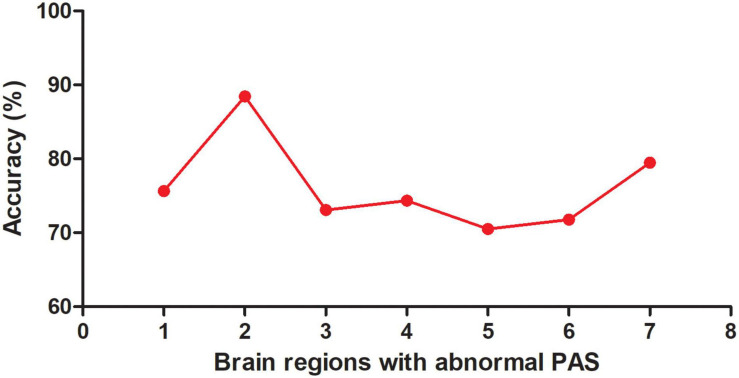
Accuracy (%) of abnormal PAS scores in a single brain region to discriminate OCD from HCs. PAS, parameter of asymmetry; OCD, obsessive-compulsive disorder; HCs, healthy controls; 1, left cerebellum Crus I; 2, bilateral middle cingulate cortex; 3, left dorsolateral prefrontal cortex; 4, right inferior occipital gyrus; 5, left inferior parietal lobule; 6, left posterior cingulate cortex; 7, left precentral gyrus/postcentral gyrus.

**FIGURE 4:**
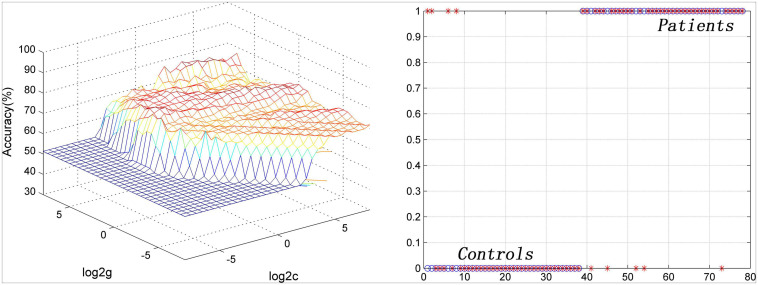
Visualization of SVM classification by using PAS scores in the bilateral middle cingulate cortex to differentiate patients with OCD from HCs. Left: 3D visualization of SVM with the best parameters; right: classification map of the PAS values of the bilateral middle cingulate cortex. SVM, support vector machine; PAS, parameter of asymmetry; OCD, obsessive-compulsive disorder; HCs, healthy controls.

Seven brain regions (1 = left cerebellum Crus I, 2 = bilateral MCC, 3 = left DLPFC, 4 = right inferior occipital gyrus, 5 = left inferior parietal lobule, 6 = left PCC, 7 = left precentral gyrus/postcentral gyrus) with abnormal PAS values were discovered in patients with OCD. SVM analysis was applied to these seven brain regions. The classification accuracies were as follows: 1 = 75.64% (59/78); 2 = 88.46% (69/78); 3 = 73.08% (57/78); 4 = 74.36% (58/78); 5 = 70.51% (55/78); 6 = 71.79% (56/78); 7 = 79.49% (62/78) (Figure 3). The results of SVM analysis showed that the PAS score of the bilateral MCC could be used to discriminate patients with OCD from the HCs with an accuracy of 88.46% (69/78), a sensitivity 87.50% (35/40), and a specificity 89.47% (34/38), respectively (Figure 4). A previous study indicated that these indicators above 80% were excellent (Gong et al., 2014). According to this criterion, the SVM result was satisfactory because all indicators were greater than 80%.

## Discussion

To the knowledge of the authors, this study was the first to explore the disrupted asymmetries of the inter- and/or intra-hemispheric FC at rest in medication-free patients with OCD by using the PAS method. Consistent with the hypothesis, this study discovered that patients with OCD showed disrupted asymmetry of the intra-hemispheric FC within (i.e., left DLPFC) and outside (i.e., left PCC, left precentral gyrus/postcentral gyrus, right inferior occipital gyrus, bilateral MCC, left IPL, and left cerebellum Crus I) the CSTC circuits. Decreased PAS in the left DLPFC was negatively correlated with Y-BOCS compulsive behavior scores in OCD. Furthermore, the exploratory SVM results suggested that decreased PAS in the bilateral MCC could discriminate patients with OCD from the HCs with appropriate accuracy, specificity, and sensitivity.

The authors’ research and other studies previously found decreased FC within the CSTC circuits at rest in OCD (Wang et al., 2018; Deng et al., 2019; Jia et al., 2020). Although these researchers found that FC strength seemed to exhibit functional asymmetry, they did not explore functional asymmetry of the interhemispheric and/or intra-hemispheric FC at rest in OCD. In the current study, the whole-brain FC was divided into inter- and intra-hemispheric FC, and the results manifested that altered FC in the brain regions within the CSTC circuits (i.e., DLPFC) might be correlated with decreased intra-hemispheric FC within the left hemisphere.

Interestingly, the results showed that decreased PAS scores in the left DLPFC were negatively correlated with the Y-BOCS compulsive behavior scores in patients with OCD. DLPFC has a core role in cognitive flexibility, set shifting, and executive planning (Kwon et al., 2009), which are all damaged in OCD (Menzies et al., 2008). The decreased intra-hemispheric FC in the left DLPFC may be related to the reduced ability to plan activities and the inability to adapt to changes in the external environment in patients with OCD (Chen et al., 2016).

Consistent with the hypothesis, the present study found altered asymmetry of intra-hemispheric FC outside the CSTC circuits in patients with OCD. These brain regions are involved in the default mode network (DMN; i.e., left PCC), executive control network (ECN; i.e., left IPL), sensorimotor network (i.e., left precentral gyrus/postcentral gyrus), and cerebellum network (i.e., left cerebellum Crus I), which might participate in the pathophysiology of OCD (Cui et al., 2020). Increased FC is regarded as a dedifferentiation and/or compensatory reallocation to the dysfunction (Guo et al., 2015). Therefore, the increased intra-hemispheric FC values in the left PCC and left precentral gyrus/postcentral gyrus in the current research may manifest an effort of compensatory reallocation to the dysfunction in the DMN and sensorimotor network at rest in patients with OCD. Decreased FC is considered as impaired coordination between brain regions (Xu et al., 2019). Thus, the reduced intra-hemispheric FC values in the bilateral MCC, left IPL, and left cerebellum Crus I suggested that the coordination was damaged within the ECN and cerebellum network. The altered intra-hemispheric FC of the DMN, ECN, sensorimotor, and cerebellum network may work together in OCD. These findings provided an additional new evidence for the pathogenesis of OCD.

In the SVM analysis of the current study, feature selection is an important step to reduce the redundancies and select meaningful features from the original feature sets (Zhang et al., 2021). Then, the remaining meaningful features were integrated into a specific classifier in an embedded manner, which was used for SVM training (Zhang et al., 2021). Classification is the approach of classifying the given input by training with an appropriate classifier (Mathew and Anto, 2017). Many researchers suggested that the SVM classifier is one of the best classifiers (Zhang et al., 2011; Menze et al., 2014). In the current study, the results of SVM analysis suggested that reduced intra-hemispheric FC of the bilateral MCC could serve as a potential neurobiological biomarker to discriminate patients with OCD from HCs, with acceptable accuracy, specificity, and sensitivity. MCC plays an essential role in response selection and cognitive task (Shima et al., 1991). Reduced intra-hemispheric FC in the bilateral MCC may be associated with difficulty in response selection between automatic habitual system and cognitive-controlled goal-directed system in patients with OCD (Dong et al., 2020).

Several innovative aspects of the current study must be mentioned. First, the division of the whole-brain FC into interhemispheric FC and intra-hemispheric FC may help explain whether abnormal FC resulted from interhemispheric FC and/or intra-hemispheric FC at rest in OCD. Second, an exploratory SVM analysis was used to investigate whether FC asymmetry could be used as a biological marker to discriminate OCD from HCs at the individual level. Third, medication-free patients with OCD were recruited, which may eliminate the potential effects of the heterogeneity of diseases and drugs on the brain FC at rest.

However, several limitations still exist. First, white and gray matters were not assessed, and their potential effects on interhemispheric FC and/or intra-hemispheric FC were unclear. Second, patients with OCD were not classified into subtypes in accordance with their clinical symptoms.

## Conclusion

In summary, the study showed disrupted asymmetry of the intra-hemispheric FC within and outside the CSTC circuits at rest in medication-free patients with OCD. The reduced intra-hemispheric FC in the bilateral MCC could be used as a biomarker to distinguish individuals with OCD from HCs. Future studies should address these limitations and further explore the clinical utility of using altered asymmetry of the intra-hemispheric FC for differentiation in OCD and prediction of treatment response (i.e., medicine, psychotherapy, and neuroregulation) in patients with OCD.

## Data Availability Statement

The original contributions presented in the study are included in the article/Supplementary Material, further inquiries can be directed to the corresponding author/s.

## Ethics Statement

The studies involving human participants were reviewed and approved by this study was approved by the Research Ethics Committee of Qiqihar Medical University. The participants were informed of the study procedures, and they signed a written informed consent. The patients/participants provided their written informed consent to participate in this study.

## Author Contributions

CJ, YO, and YC engaged in data analysis and wrote the manuscript. PL and WG designed and conducted the study. JM, CZ, DL, RY, TS, LS, YW, GZ, ZS, WW, and XW participated in patient assessment and imaging data collection. All authors contributed to the article and approved the submitted version.

## Conflict of Interest

The authors declare that the research was conducted in the absence of any commercial or financial relationships that could be construed as a potential conflict of interest.
