# Supplementary material for: Disrupted Asymmetry of Inter- and Intra-Hemispheric Functional Connectivity at Rest in Medication-Free Obsessive-Compulsive Disorder
Source: Front Neurosci. 2021 Jun 9;15:634557. doi: 10.3389/fnins.2021.634557 (PMC8220135; doi:10.3389/fnins.2021.634557)
Supplement: Supplementary file 1 [file Data_Sheet_1.docx]

Supplementary Material

# Supplementary Tables

**Table S1** PAS values in OCD and HCs

| Cluster location | OCD  (n =40) | HCs  (n = 38) | *t* | *p* |
| --- | --- | --- | --- | --- |
|  |  |  |  |  |
| Left Posterior Cingulate Cortex | -0.2317±0.4818 | -0.9592±0.5017 | 6.533 | <0.001 |
| Left Precentral Gyrus/Postcentral Gyrus | -0.0511±0.3778 | -0.5095±0.3810 | 5.334 | <0.001 |
| Right Inferior Occipital Gyrus | -0.0012±0.5786 | -0.6103±0.5315 | 4.835 | <0.001 |
| Left Dorsolateral Prefrontal Cortex | -0.7883±0.5424 | -0.1271±0.4628 | -5.777 | <0.001 |
| Bilateral Middle Cingulate Cortex | -0.9633±0.4091 | -0.4561±0.4291 | -5.344 | <0.001 |
| Left Inferior Parietal Lobule | -0.7573±0.4351 | -0.2032±0.4966 | -5.248 | <0.001 |
| Left Cerebellum Crus I | -0.7294±0.4475 | -0.0704±0.7059 | -4.950 | <0.001 |

PAS = parameter of asymmetry; OCD = obsessive-compulsive disorder; HCs = healthy controls

# Supplementary Figures


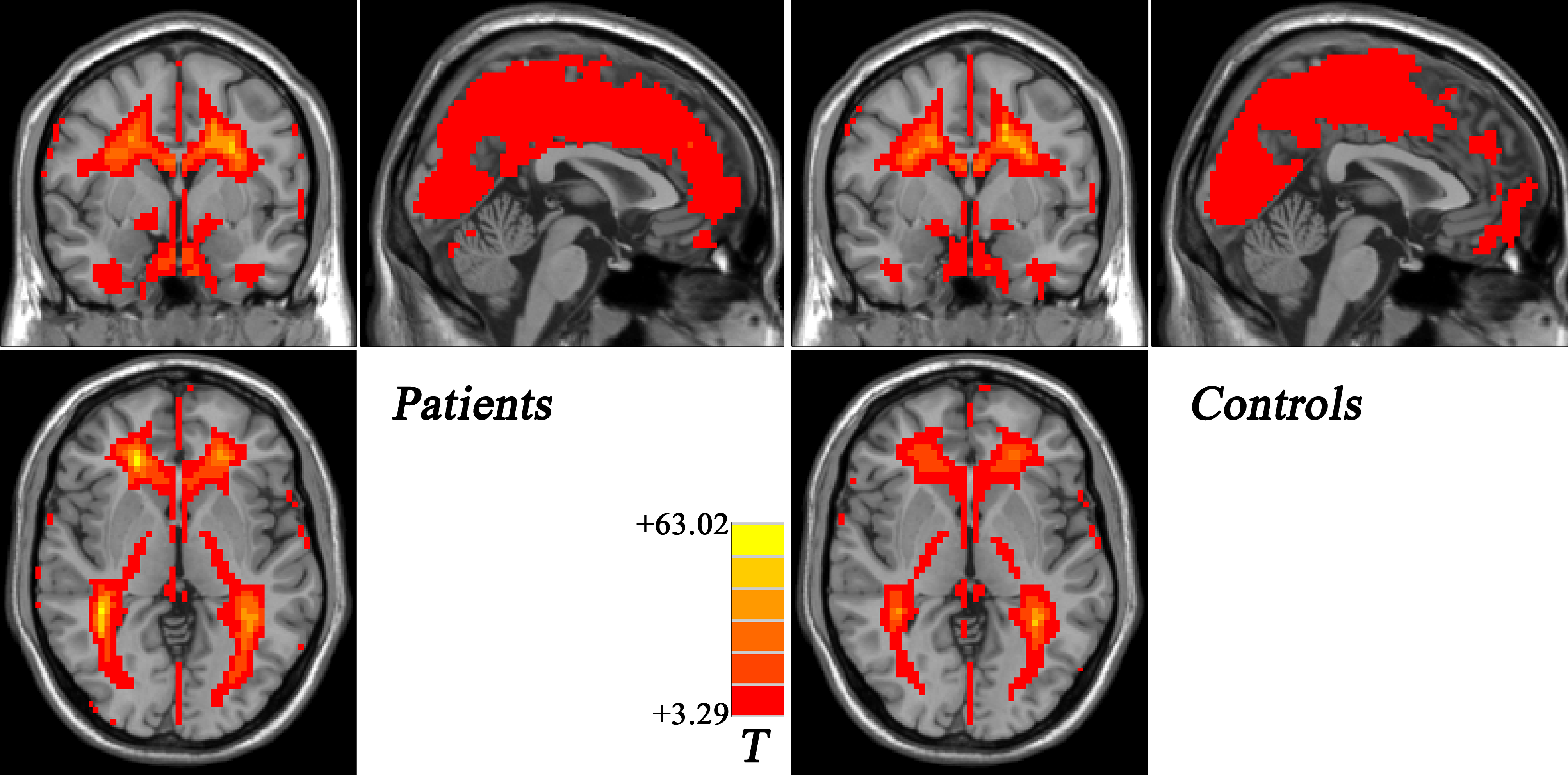


Figure S1. The results of one-sample *t*-tests of PAS in each group.
